# Pattern of peer review proforma of medical journals of Pakistan

**DOI:** 10.12669/pjms.35.4.713

**Published:** 2019

**Authors:** Faaiz Ali Shah, Mian Amjad Ali, Zahid Nazar, Haroon Ur Rasheed

**Affiliations:** 1 Dr. Faaiz Ali Shah, FCPS. Department of Orthopaedics & Traumatology, Lady Reading Hospital Peshawar KP, Pakistan; 2 Dr. Mian Amjad Ali, PhD. Department of Orthopaedics & Traumatology, Lady Reading Hospital Peshawar KP, Pakistan; 3 Dr. Zahid Nazar, FCPS. Department of Psychiatry, Lady Reading Hospital Peshawar KP, Pakistan; 4 Dr. Haroon Ur Rasheed, MBBS. Department of Orthopaedics & Traumatology, Lady Reading Hospital Peshawar KP, Pakistan

**Keywords:** Analysis, article, journal, manuscript, peer review, publication

## Abstract

**Objective:**

To analyze the contents and format of peer review proforma of Medical journals of Pakistan.

**Methods:**

This descriptive study was conducted in the Department of Orthopaedics and Traumatology Lady reading Hospital Peshawar Pakistan from 3^rd^ August 2018 to 9^th^ February 2019.An email was sent to the chief editors of all the medical journals listed on the official website (www.pmdc.org.pk) of Pakistan Medical and Dental Council (PM&DC).They were requested to send peer review proformas of their journals. The received proformas were analyzed for major contents and format or style. The proforma had a structured format when each portion of the manuscript i.e, title, abstract, key words, methodology, results, discussion, conclusion and references were individually sectioned for evaluation. Whereas in the unstructured proformas the reviewer was asked to assess the manuscript as a whole.

**Results:**

We received 41 proformas via emails. Majority (82.9%) of the proformas were structured while 17% were unstructured. A scoring or rating system for the manuscript was present in 31.7% of the proformas while 43.9% of the proformas were without any scoring system. Guidelines for the peer reviewers were given in 58.5% of the proformas. The peer review policy (closed or open) was mentioned in only 7.3%.About 9.7% of the proformas asked the reviewers to disclose conflict of interests.

**Conclusion:**

A spectrum of contents and format of peer review proformas of medical journals were observed. We found structured peer review proforma with a scoring scale comprehensive and more appropriate for peer review.

## INTRODUCTION

The process of analyzing a research manuscript or a project by third party experts or peers for publication or allocation of grant is called peer review.^1^ Although peer review process is not free from flaws,^2^ yet it is the most common and popular method to assess research.^3^ In the present scientific world peer review is considered as a “gold standard”^4^ or “cornerstone of quality assurance”^5^ of scientific publications. The scientific community accept a hypothesis only when published in a peer reviewed medical journal.^6^ Peer reviewed journals are the only journals that are considered for impact factor by the Institute for Scientific Information (*ISI*).^2^ The authors consider publication in a peer reviewed journal prestigious.^7^ Peer review proforma or form is a manuscript assessment tool sent by editors to the peer reviewers along with the manuscript. Every journal has its unique peer review proforma formulated in accordance with its peer review policy. The peer reviewer evaluate the manuscript according to the proforma and send it back to the editor. This feedback helps the editor to decide the fate of the manuscript.^8^ Surprisingly the assessment of peer review quality, standard and consistency is difficult because exact data of peer review is not yet available.^9^,^10^ Moreover the study of peer review is difficult and complex.^9^ The same manuscript receives different ratings by different peer reviewers evaluated through the same proforma resulting in low inter-rater reliability.^11^,^12^ Furthermore peer reviewers were found unable to point out all the errors in the manuscript.^13^

Since there is a growing need for systemic studies on peer review,^14^ we plan that one aspect of peer review assessment is to analyze the peer review proforma. By improving the peer review proforma quality of the manuscript evaluation can be improved. Unluckily we could not find a single study on this subject in Pakistan but our intention to improve peer review process compelled us to conduct this study. The scope and policies of peer review varies from journal to journal and a uniform peer review proformas for all the journal will not be possible. The aim of our study was to stress the importance of a good quality comprehensive peer review proforma and not to undermine or advocate any particular peer review proforma of a journal. We are confident that this study will provide a foundation for further large scale studies on this topic in Pakistan.

## METHODS

This descriptive study was conducted in the Department of Orthopaedics and Traumatology Lady Reading Hospital Peshawar Pakistan from August 2018 to February 2019. All the medical journals listed on the official website (www.pmdc.org.pk) of Pakistan Medical and Dental Council (PM&DC) were included in the study. An email was sent to the chief editors of these medical journals requesting them to send their peer review proforma for study purpose. They were assured that the names of their journals will be kept strictly confidential and the individual proformas will not be made public. After the receipt of peer review proformas, each proforma was analyzed for major contents and format or style. By format (style) of the proforma we mean structured and unstructured. The proforma was considered structured if each portion of the manuscript i.e, title, abstract, key words, methodology, results, discussion, conclusion and references were individually mentioned for evaluation in the proforma. In the unstructured proformas the reviewer was required to assess the manuscript as a whole.

All the data variables of the proforma were analyzed with SPSS (version 20). Frequency, Percentages and *P* values of the important variables were calculated. A *P* value less than 0.05 was considered significant. Data presented in table where necessary.

## RESULTS

There were 72 medical journals listed on the official website of Pakistan Medical and Dental Council (PM&DC). We sent an email to the chief editors of all journals for peer review proforma.We received 41 proformas (33 general and 8 specialty journals).The salient features of the proformas are summarized in Table-I. Most (41.4%, n=17) proformas consisted of only two pages while 12(29.2%) proformas had three, 10 (24.3%) had one page and 4(9.7%) proformas had four pages. Structured proformas with scoring system had maximum number of pages (3 or 4).We found that although majority (82.9 n=34) of the proformas were structured still a good percentage (17%,n=7) were unstructured.(*P* value 0.00001) Most(43%, n=18) of the proformas were without any proper scoring or rating system. (*P* value 0.230)The scoring system and scoring scale was variable. The scoring system for individual components of the manuscript (title, abstact, methodology, results, conclusion, references) was found in 9(69.2%) proformas while scoring for overall manuscript was noted in only 4(30.7%). Scoring scale of 4(30.7%)proformas were 1 to 4(excellent, good, average, poor) while a soring scale of 1 to 5 was found in 3(23%), 0 to 5 in 2(15.3%) 0 to 10 in 2(15.3%) and 1 to 10 in 2(15.3%) proformas. Manuscript evaluation by only ticking Yes or No and without any scoring was noted in 7(38.8%) proformas while a combination of ticking Yes or No and overall scoring was observed in two proformas. Global rating (e.g, advised revision, approved, rejected or accepted without changes, with minor changes, with major changes etc) of the manuscript varied from journal to journal but included in the majority (51.2%,n=21) of proformas.

**Table I:** The salient features of peer review proformas of medical journals of Pakistan.

S.No	Proforma Content/format	Mentioned		Not mentioned		Z value	P value

		Number of Journals	Percentages	Number of Journals	Percentages		
1	Journal name	33	80.4	8	19.5	6.0	0.00001
2	Reviewer’s information/identification	34	82.9	7	17	7.9	0.00001
3	Guidelines/instructions for reviewers	24	58.5	17	41.4	0.9	0.368
4	Reviewer’s conflict of interest	4	9.7	37	90.2	12.9	0.00001
5.	Proforma structured	34	82.9	7	17	7.9	0.00001
6	Scoring/Rating of manuscript	13	31.7	18	43.9	1.2	0.230
7	Type of manuscript	16	39	25	60.9	1.93	0.053
8	Type of peer review	3	7.3	38	92.6	14.7	0.00001
9	Reviewer’s signature	20	78.7	21	51.2	0.27	0.78
10	Priority of the manuscript for publication	2	4.8	39	95.1	20.2	0.00001
11	Reviewer’s willingness for revision of manuscript	3	7.3	38	92.6	15.0	0.00001
12	Method of sending back peer review proforma	7	17	34	82.9	7.9	0.00001

Only 1(2.4%) proforma had asked the reviewers to comment on Hypothesis statement and Research question. Majority (60.9%,n=25%) of the journals had not mentioned the type(original article, review article, case report etc) of the manuscript they want to review nor did they asked the reviewers to classify the manuscript (*P* value 0.053).Open peer review was mentioned in 2 (66.6%) proformas and single blind peer review in 1 (33.3%) while majority (92.6%,n=38) of the journals did not mention peer review type in their proformas (*P* value 0.00001).Only two (4.8%) journals had asked from the reviewers the priority(immediate/routine) of manuscript for publication if accepted by the editor for publication(*P* value 0.00001).Peer review proformas of two medical journals(one general and one specialty journal) were exactly the same in contents and format. A small percentage (17%) of proformas had instructions for the peer reviewers regarding the method of sending back the filled proformas to the editor. The proformas of 5(71.1%) journals directed the peer reviewers to send back the proforma via email while only 2(28.5%) journals demanded paper filled proforma via postage.

## DISCUSSION

In our study we found that majority(41.4%) of the peer review proformas had no instructions or guidelines for peer reviewer to review the manuscript.(*P* value ≥ 0.05) In Pakistan there are no proper training or published guidelines and protocols on how to perform a good peer review and reviewers usually learnt peer review skills by themselves. Therefore we strongly recommend inclusion of guidelines for reviewers in every journal’s proforma to ensure compliance and consistency in peer review. The global situation is not much different than our country as Warne^15^ has rightly pointed out that 32% peer reviewers are trained in peer reviewing by reading guidelines given by the journal, 18% by reading guidelines of Committee on Publication Ethics (COPE),19% by their supervisors and 16% by their colleagues. A declaration of the peer reviewer as to the possible conflict of interest while reviewing the manuscript was not mentioned in vast majority(90.2%) of the peer review proformas (*P* value < 0.05).The policy of disclose of conflict of interests by the journals are variable. A study of 37 ophthalmology journals noted that 100 percent of the journals required the authors to declare conflict of interest,30% demanded the editors to declare conflict of interests and 60% asked the reviewers to disclose conflict of interests.^16^ Peer reviewer’s evaluation of a manuscript can have an element of bias if conflict of interest exists resulting in undermining the trust of authors in the peer review process.^17^ To prevent bias in review and to promote good quality peer review conflict of interest statement must be included in every peer review proforma.

We observed that majority(82.9%) of the proformas were structured while 17% were un structured(*P* value < 0.05).Research has shown that peer reviewers have a very low level of inter rated reliability and different reviewers concentrate on diverse issues while assessing a manuscript.18,19 It is also true that peer review is actually a judgment and judgment of different peer reviewers about the same manuscript can be different.7 In our opinion the evaluation of a manuscript with unstructured proforma is more subjective and inconsistent in nature. The subjective review can be enhanced with more objective criteria. We proposed that with a structured proforma and scoring system these deficiencies can be minimized as the reviewer will have to concentrate equally on all aspects of manuscript and weightage given to each component individually. The unstructured proforma may be more suitable to assess qualitative research and the opponents can argue that with a unstructured proformas the peer reviewers are more free to judge and comment but the possibility of harsh or aggressive comments by some of the reviewers can not be avoided.

We noted that most (43.9%) of proformas had no scoring or rating system of the manuscript while it was mentioned only in 31.7% of proformas (P value ≥0.05). Moreover the scoring scale was different in different proformas. A peer review report is considered low quality report if it lacks a rating or scoring system.^12^,^20^ Interestingly one peer review proformas of an impact factor journal was neither structured nor had a scoring system, the other was structured but devoid of a scoring system and the third one had both a structured format and a scoring system.

The peer review type was not mentioned in majority (92.6%) of the proformas (*P* value < 0.05). Open peer review policy was mentioned in two proformas and single blind in one proforma. There are two essential types of peer review:^21^ Closed and open. In the closed peer review identification of one party (usually the reviewer) is not disclosed. Closed review can manifest in single blind review in which the authors don’t know the reviewers but reviewers know author’s identification.

Majority of biomedical journals follow this model. In the double blinded review both the parties (reviewers and authors) do not know each other. Double blinded peer review is followed by most of the nursing journals. In open peer review the identity of reviewer and author is known to each other. Many reputable medical journals like British Medical Journal (BMJ) follow open peer review policy. Each review type has advantages and disadvantages.^22^ In a peer review survey by Ware^23^ 56% authors preferred double blinded peer review, 25% single blind, 13% open peer review and 5% preferred post publication review. The same survey also noted that the most effective peer review was double blind (71% respondents) followed by single blind (52%), post publication(37%) and open peer review (26%). Besides these traditional peer review models hybrid peer review, priori peer review, posteriori peer review and other less popular forms of peer reviews are also being practiced in scholarly world.^24^

In our study we documented that majority(82.9%) of the proformas had no instructions for the reviewers for sending back the proformas (*P* value < 0.05).Five journals directed the peer reviewers to send back the filled proforma via email and two journals demanded paper filled proforma via postage. About 30% of peer reviewers of one Pakistani impact factor medical journal submit their reports on line.^25^ The journal prefer electronic submission of peer review reports because it is not only fast and economically feasible but the overall peer review process take less time to complete. A good peer review proforma must act as a filter for the manuscript. It must have instructions for the reviewers and conflict of interest statement. It must be structured with a scoring system. It should facilitate the authors to submit rather than discouraging them. The authors must be provided a mechanism to rate the peer review report of their manuscript. The authors should have insight of peer review process to relieve their anxiety and restore their confidence in improving the quality of their manuscript. The development of peer review skills will need uniform guidelines, regular workshop and mentorship opportunities specially to young researchers. Peer reviewers must be recognized or rewarded. And last but not the least we must not abandon the peer review rather improve it.

Our study was not an in-depth analysis of all the peer review proforomas. We concentrated only on the salient features of the proformas. It is possible that many important contents might be missed. More over all the peer review proformas were analyzed by the principal author only and the possibility of error in the analysis can not be omitted. We can not claim that our study covered all the issues of the peer review proformas because of the unavailability of all the proformas (specially journals covering basic medical sciences). Furthermore, from proformas we could not assessed whether it is used for evaluating pure quantitative or qualitative research or both. We therefore, recommend well designed studies with a larger sample size on this topic to clearly answer these questions.

## CONCLUSION

A spectrum of contents and format of peer review proformas of medical journals were observed. We found structured peer review proforma with a scoring scale comprehensive and more appropriate for peer review.

### Authors Contribution

**FAH:** Conceived, designed, manuscript writing, statistical analysis.

**MAA, ZN:** Did review and final approval of manuscript.

**HUR:** Did data collection.
